# Evaluation of a Rapid Kit for Detection of IgM against *Leptospira* in Human

**DOI:** 10.1155/2019/5763595

**Published:** 2019-02-07

**Authors:** Mohan Rao, Fairuz Amran, Nadia Aqilla

**Affiliations:** Bacteriology Unit, Institute for Medical Research, Ministry of Health Malaysia, Jalan Pahang, 50588 Kuala Lumpur, Malaysia

## Abstract

**Introduction:**

Leptospirosis is an acute febrile illness, known for its protean clinical manifestations and the challenge in differentiating from other infectious diseases. Standardized confirmatory test is antibody dependent and not accessible by the suburban community. This study measures efficiency of an immune-chromatographic assay, Leptocheck WB, in detecting acute leptospirosis.

**Methods:**

A total of 142 sera were used for kit evaluation. Sensitivity, specificity, positive predictive value (PPV), and negative predictive value (NPV) were calculated by comparing rapid kit results with gold standard laboratory, microscopic agglutination test (MAT).

**Results:**

We found this rapid kit to have a sensitivity and specificity of 66.6% and 78.9%, respectively, whereas the PPV and NPV of the kit appeared to be 73.3% and 73.2%, respectively.

**Discussion:**

Test efficiency of this rapid kit is reasonable. It is specific in detecting leptospiral antibody and assures clinician of accurate diagnosis by having higher PPV and NPV. It is prompt and efficient in comparison with conventional methods in assisting differential diagnosis. High sensitivity and specificity leptospirosis rapid test is indeed a crucial measure to assist the diagnosis of acute undifferentiated febrile illnesses.

## 1. Introduction

Acute febrile illness is a clinical syndrome, contributes to substantial morbidity and mortality worldwide [1]. The epidemiology of infectious cause varies from one country to another country [2, 3]. Leptospirosis is underrecognized but the common cause of acute febrile illness [4]. Its global distribution ranging from 10 to 100 human cases per 100000 individuals with higher incidence is commonly found in tropics and subtropics [5].

Leptospirosis is caused by pathogenic species of the spirochete *Leptospira*. It is known for its protean manifestations, from being asymptomatic to a severe disease with multiple organ failure that requires intensive care approach [6]. High fever, headache, chills, arthralgia, myalgia, jaundice, diarrhea, nausea, and vomiting are common symptoms manifested by the illness [7]. Often times, it is difficult to differentiate, due to its nonspecific clinical manifestations and poor background knowledge on infectious epidemiology [8, 9]. Failure to recognize leptospirosis delays antibiotic administration and may lead to poor outcomes [10].

Confirmatory laboratory test is very crucial for optimal treatment and management of the illness. Microscopic agglutination test (MAT), organism DNA detection via nucleic acid amplification test (NAAT), enzyme-linked immunosorbent assay (ELISA), and organism isolation via culture method are a few tests performed in our reference laboratory. However, there are limitations to their use and interpretation of the results. These tests are tedious, sophisticated, time-consuming, and operator dependent; therefore, they are not suited for early diagnosis [11]. Hence, a timely manner diagnosis in conjunction with laboratory support such as rapid test is often preferred in the hospital environment. Any efforts in developing a novel diagnostic kit for rapid diagnosis of leptospirosis are appreciable, valuable, and significant. Early diagnosis is possible by having an ideal laboratory support that is rapid, sensitive, safe, simple, and economical [12].

Leptocheck WB is a bedside, rapid, and qualitative diagnostic kit used for preliminary diagnosis of leptospirosis. It utilizes the principle of agglutination of antibodies with respective antigen in immunochromatography format along with the use of nanogold particles as agglutination revealing agent. As the serum flows through the membrane assembly of the kit, agglutinating sera for human IgM-colloidal gold conjugate forms complexes with IgM antibodies in the sample. As these complexes move further on the membrane, they will be immobilized by *Leptospira* genus specific antigens coated on the membrane, leading to the formation of red- to purple- coloured band at the region “T” of the kit indicating presence of *Leptospira* IgM antibodies.

We have evaluated a solid-phase commercial gold immunochromatographic assay kit (ICA), which was manufactured by Zephyr Biomedicals, Goa, India. This kit was evaluated for its efficacy identifying *Leptospira* antibodies in acute sera, plasma, or blood for rapid bedside screening.

## 2. Materials and Methods

This diagnostic kit evaluation was conducted in Reference Laboratory for Leptospirosis, Institute for Medical Research (IMR), Kuala Lumpur. It was performed by trained laboratory technician.

### 2.1. Microscopic Agglutination Test

The current gold standard for the laboratory diagnosis of leptospirosis is the microscopic agglutination test (MAT). MAT was performed on all the sera using a panel of 20 serovars recommended by the WHO Collaborative Centre for Leptospirosis and 6 local isolates. The reference strains obtained from the WHO Collaborative Centre for Leptospirosis, Amsterdam are Australis, Autumnalis, Bataviae, Ballum, Canicola, Grippotyphosa, Icterohaemorrhagiae, Javanica, Pomona, Pyrogenes, Tarrasovi, Sejroe, and Patoc [5, 13].

### 2.2. Sera/Plasma Selection

A total of 142 sera were used in this evaluation. Among these sera, 66 were positive and 76 were negative. Thirty-one paired samples (62 sera) were gathered to test the efficiency in detecting IgM of *Leptospira*; however, these sera were not included in the diagnostic performance calculation. Sera were considered positive if the MAT titer was ≥400 or with 4-fold rise in titer detected between acute (≤7 days of illness) and convalescent sera (≥7 days of illness) from the same patient. Meanwhile, sera were considered negative if MAT titer is ≤ 50, indicating other infective causes [14]. These are retrospective sera of patients recruited from various hospitals localized in Federal Territory, Selangor and Pahang State, Malaysia. They were sera sent to IMR for laboratory diagnosis of leptospirosis.

An additional 50 sera, tested positive for other infectious diseases, were obtained from the serology laboratory in Institute for Medical Research. Ten positive sera, respectively, from melioidosis, Brucellosis, Rickettsioses, dengue NS1 positive, and dengue IgM were used to assess the cross-reactivity status of the ICA. These sera were not included in the study of diagnostic performance.

### 2.3. Procedure of Test

**Figure 1:**
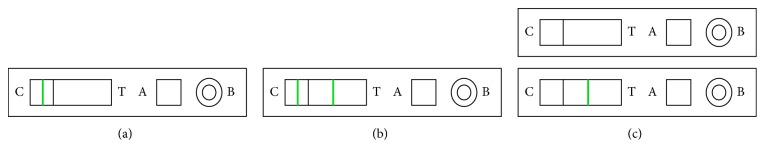
The summary for test procedure and interpretation of test results. (a) Negative result: only one coloured band appears in the control window “C.” (b) Positive result: in addition to the control band, another red to deep purple coloured band appears in the test window “T.” (c) Invalid result: the test should be considered invalid if no bands appear on the device. The test should also be considered invalid if only test band appears and no control band appears. Repeat the test with a new device ensuring that the test procedure has been followed accurately.

Leptocheck WB, Zephyr Biomedical, India, is a rapid test kit evaluated in this study. According to the manufacturer, this kit is designed for the detection of the acute-phase antibody, IgM. The principle of the test is based on the immunochromatographic agglutination of circulating antibodies with specific antigen using nanogold particles as the agglutination revealing agent. Samples in the form of sera, plasma, or blood were tested on the evaluation kit as per instruction by the manufacturer. Result interpretation is solely based on the appearance of the red band within designated time.  Figure 1 explains the subjective visual interpretation of the test kit.

### 2.4. Data Analysis

Diagnostic performance was calculated by comparing ICA results with MAT of selected sera by tabulating the results in a crosstab formula. True positive, true negative, false positive, and false negative results were identified. The sensitivity, specificity, positive predictive value (PPV), and negative predictive value (NPV) of the diagnostic kit were calculated using the following formulas:

(1) $$\begin{array}{c} \text{sensitivity}\left(\%\right)=\frac{\text{true positives}}{\left(\text{true positives}+\text{false negatives}\right)}\times 100,\\ \text{specificity}\left(\%\right)=\frac{\text{true negatives}}{\left(\text{false positives}+\text{false positives}\right)}\times 100,\\ \text{positive predictive value }\left(\%\right)=\frac{\text{true positive}}{\left(\text{true positive}+\text{false positive}\right)}\times 100,\\ \text{negative predictive value}\left(\%\right)=\frac{\text{true negative}}{\left(\text{false negative}+\text{true negative}\right)}\times 100\%. \end{array}$$

## 3. Results

In this study, 44 of 66 positive leptospirosis cases were tested positive using the ICA giving a case sensitivity of 66.6%. Sixty of 76 negative leptospirosis cases were tested negative for ICA, resulting in a specificity of 78.9%. The positive predictive value (PPV) and the negative predictive value (NPV) of the assay were 73.3% and 73.2%, respectively. Results are as tabulated in Tables 1 and 2. In terms of cross-reactivity evaluation, minimal cross activities were observed with dengue and melioidosis sera at a percentage of 10% and 20%, respectively (Table 3), whereas 14/31 (45%) acute sera among the paired samples were positive using ICA; albeit MAT titer were either negative or inconclusive.

## 4. Discussion

Diagnosis of acute febrile illness is difficult in tropical settings where many possible etiologies can be responsible for the illness. The urgency for rapid diagnosis of leptospirosis is necessary for accurate antibacterial therapy and to evade impending complications [6]. Commonly, leptospirosis remained underrecognized or misdiagnosed due to their paradoxical clinical manifestations. Molecular-based methods involving real-time PCR have been successful in providing rapid diagnosis [15]; however, its requirements for specialized personnel, sophisticated equipment, and expensive rather made it harder for urgent diagnosis.

In our evaluation, the test efficiency of this immunochromatographic assay was generally lower than those reported previously [16, 17, 18]. This rapid kit was found to be moderately sensitive and specific with acceptable PPV. Its performance was particularly moderate for samples collected within first week of illness. Unfortunately, this time interval is crucial for therapeutic decision.

Although the sensitivity of the kit is lower than the specificity, this could be reasoned by no available or inadequate antibody in the acute sera/early phase of infection [19]. It is beyond the capability of the kit in detecting low-level antibody. Therefore, it is advisable to repeat the test after the period of seroconversion. On the contrary, the antigen used in this agglutination principled test plays a major role in estimating the efficiency of the test. Negative test on serial samples does not rule out the probability of the infection since the choice of “antigen coverage” coated in the kit (not disclosed by the manufacturer due to confidentiality) is probably not within our local spectrum, resulting in false negative outcome [20].

In this study, we discovered that the kit has reasonable PPV and NPV. These findings allow clinicians to narrow down the diagnosis as this kit is specific in detecting *Leptospira* IgM antibody. Therefore, it encourages initiation of appropriate therapy without a delay [10]. As the specificity of this test kit is moderate, it permits minimal cross-reactivity with other acute febrile illnesses. Hence, it is particularly difficult to determine the actual diagnosis and can be only confirmed via culture or polymerase chain reaction to detect the DNA of the respective bacteria or virus. However, this approach is deemed not possible as many peripheral laboratories are not equipped to do so.

Other than providing reasonable results, this test kit has several other advantages. In comparison with tedious and time-consuming gold standard MAT and ELISA test [11], this test kit is rapid in providing result within given time scale, simple to perform, without the need of sophisticated equipment. It can be performed at bedsides, in the laboratories, or at the field. The test can be used in resource-poor settings, by investigators with only limited training [12]. However, MAT and ELISA remained as the gold standard laboratory diagnosis despite being tedious and sophisticated due to their vast antigen coverage from different serogroups and serotypes, serial dilution of sera to appreciate seroconversion and to overcome the prozone effect.

In view of multiple cause of acute febrile illness, patients' medical history and clinical assessment are not sufficient in providing absolute diagnosis. Screening test that is sensitive and specific in the acute phase of infection would be of great benefit for health care. In reemerging zoonosis, a rapid diagnosis of leptospirosis would help physicians in providing appropriate treatment.

## Figures and Tables

**Table 1:** Test results using the Leptocheck WB *Leptospira* rapid test kit against MAT (gold standard).

		Microscopic agglutination test (MAT)		
		Positive	Negative	Total
ICA Leptocheck WB *Leptospira* rapid test kit	Positive	44 (true positive)	16 (false positive)	60
	Negative	22 (false negative)	60 (true negative)	82
	Total	66	76	142

**Table 2:** Sensitivity, specificity, positive predictive value, and negative predictive value of the ICA Leptocheck WB *Leptospira* rapid test kit against MAT (gold standard).

Statistical measurement	Test efficiencies (%)
Sensitivity	66.6
Specificity	78.9
PPV	73.3
NPV	73.2

**Table 3:** Cross reactivities of the Leptocheck WB *Leptospira* rapid test kit with other causes of acute febrile illnesses.

Serum samples tested positive serologically to other infectious diseases	Leptocheck WB rapid test for IgM antibodies to *Leptospira*		Percentage of cross-reactivity between immunochromatographic tests with other infectious diseases (%)
	Positive	Negative	
Dengue (IgM positive)	1	9	10
Dengue (NS1 positive)	1	9	10
Melioidosis	2	8	20
Brucellosis	0	10	0
Rickettsioses	0	10	0

## Data Availability

The data used to support the findings of this study are available from the corresponding author upon request.
